# RRM2 Alleviates Doxorubicin-Induced Cardiotoxicity through the AKT/mTOR Signaling Pathway

**DOI:** 10.3390/biom12020299

**Published:** 2022-02-12

**Authors:** Yuheng Jiao, Yanyan Li, Jiayan Zhang, Song Zhang, Yafang Zha, Jian Wang

**Affiliations:** 1 Department of Cardiology, Renji Hospital, School of Medicine, Shanghai Jiao Tong University, 160 Pujian Road, Shanghai 200127, China; drjyh97@163.com (Y.J.); zhangjiayan77@gmail.com (J.Z.); zyf19121708287@163.com (Y.Z.); 2837985@sjtu.edu.cn (J.W.); 2 Department of Cardiology, Xinhua Hospital, School of Medicine, Shanghai Jiao Tong University, 1665 Kongjiang Road, Shanghai 200092, China; yannibest@126.com

**Keywords:** RRM2, doxorubicin, AKT/mTOR pathway, cardiotoxicity

## Abstract

Doxorubicin (DOX) is an effective chemotherapeutic agent that plays an unparalleled role in cancer treatment. However, its serious dose-dependent cardiotoxicity, which eventually contributes to irreversible heart failure, has greatly limited the widespread clinical application of DOX. A previous study has demonstrated that the ribonucleotide reductase M2 subunit (RRM2) exerts salutary effects on promoting proliferation and inhibiting apoptosis and autophagy. However, the specific function of RRM2 in DOX-induced cardiotoxicity is yet to be determined. This study aimed to elucidate the role and potential mechanism of RRM2 on DOX-induced cardiotoxicity by investigating neonatal primary cardiomyocytes and mice treated with DOX. Subsequently, the results indicated that RRM2 expression was significantly reduced in mice hearts and primary cardiomyocytes. Apoptosis and autophagy-related proteins, such as cleaved-Caspase3 (C-Caspase3), LC3B, and beclin1, were distinctly upregulated. Additionally, RRM2 deficiency led to increased autophagy and apoptosis in cells. RRM2 overexpression, on the contrary, alleviated DOX-induced cardiotoxicity in vivo and in vitro. Consistently, DIDOX, an inhibitor of RRM2, attenuated the protective effect of RRM2. Mechanistically, we found that AKT/mTOR inhibitors could reverse the function of RRM2 overexpression on DOX-induced autophagy and apoptosis, which means that RRM2 could have regulated DOX-induced cardiotoxicity through the AKT/mTOR signaling pathway. In conclusion, our experiment established that RRM2 could be a potential treatment in reversing DOX-induced cardiac dysfunction.

## 1. Introduction

Doxorubicin, a broad-spectrum chemotherapeutic drug, is commonly used in the clinical treatment of cancer, with a potent curative effect [1]. However, promoting DOX usage in clinical treatment still faces many obstacles, such as severe cardiotoxicity and heart failure [2,3]. It is believed that many factors are involved in its cardiotoxicity, such as the excessive accumulation of reactive oxygen species (ROS), calcium dysregulation, and disturbance of the apoptosis and autophagy pathways [4,5,6,7,8]. Current evidence has shown that the excessive accumulation of ROS is the culprit of DOX-induced cardiomyopathy, resulting in irreversible cardiac dysfunction [9]. However, some evidence has demonstrated that DOX could lead to apoptosis without inducing ROS production and oxidative stress [10]. DOX-induced cardiotoxicity can be alleviated by inhibiting cardiomyocyte apoptosis [11]. Accordingly, targeting the above mechanisms provides an effective therapeutic direction to alleviate DOX-induced cardiotoxicity.

Ribonucleotide reductase (RR) plays a crucial role in DNA synthesis and can limit its repair [12]. The ribonucleotide reductase regulatory subunit M2 (RRM2), a part of nucleotide reductase, catalyzes the inhibition of ribonucleotides, yielding deoxyribonucleotides [13]. Previous studies have revealed that RRM2 plays a vital role in promoting cell proliferation, migration, and invasion, while inhibiting cell apoptosis [14]. In addition, RRM2 could affect cell proliferation—specifically, inhibition of the cell cycle, which is manifested as the apoptosis of human neuroblastoma cells [15]. Moreover, RRM2 expression can be used as an indicator to predict responses to chemotherapy [16,17]. Another study showed that by inducing the overexpression of Rrm1/Rrm2 in rats, the content of RNA enzyme was significantly increased, which improved cardiac function without causing cardiac remodeling in infarcted rats [18]. However, the roles and mechanisms of RRM2 in DOX-induced cardiotoxicity are still unclear.

Collectively, our findings indicated that RRM2 could alleviate DOX-induced myocardial damage, possibly by inhibiting excessive cardiomyocyte apoptosis and autophagy. The results demonstrated that RRM2 could provide a novel therapeutic direction to ameliorate DOX-induced cardiotoxicity.

## 2. Materials and Methods

### 2.1. Animals and Treatment

Male C57/B6 mice were purchased from Jihui Laboratory Animal Breeding Co., Ltd. (Shanghai, China). All animals were kept in an SPF barrier environment at a constant temperature of 20°–25° and were provided with sufficient food and water. After dissolving DOX with saline, the mice were prepared to receive the intraperitoneal injection. The experimental group was injected with DOX (i.p. 15 mL/kg), while the control group was injected with the same dose of saline. Five days later, the heart tissue sample was collected under the premise of using isoflurane anesthesia.

### 2.2. Cell Studies

Neonatal primary cardiomyocytes from mice were isolated according to previous studies [19]. The cardiomyocytes were kept in DMEM supplemented with 10% fetal bovine serum (Sigma, Saint Louis, MO, USA) and 1% penicillin-streptomycin solution (Hyclone, Logan, UT, USA). The cells were treated with DOX (1 µmol/L) in the medium for 24 h. In addition, RRM2 inhibitor (MCE, New Jersey, USA) and AKT/mTOR inhibitors (MCE, New Jersey, USA) were added into the medium, respectively.

According to the product instructions of Zorin, the small interference targeting RRM2 (si-RRM2) was transfected into cardiomyocytes for 12 h using Lipo3000. After that, we measured RRM2 protein and mRNA levels to detect the silencing efficiency of si-RRM2. 

### 2.3. Recombinant Adenovirus Was Delivered to Mice Ventricular and Primary Cardiomyocytes

The adenovirus with RRM2 overexpression (Ad-RRM2) and control virus (Ad-GFP) were constructed by Hanbio Biotechnology, China. Mice were anesthetized by isoflurane followed by continuous inhalation of isoflurane using a mask oxygen inhalation device. During exposure to the heart at the strongest apical beat, Ad-RRM2 or Ad-GFP (50 μL) was injected into the 4–5 positions on the left ventricular wall using a disposable sterile syringe. Four days later, subsequent treatment was carried out.

The primary cardiomyocytes were incubated in a 6-well plate, and the virus was transfected into cells. Replacing the new medium 12 h after transfection was necessary, and the subsequent treatment was carried out after 24 h.

### 2.4. Cell Proliferation Assay

To further verify the cell viability, a cell count kit-8 (CCK-8, Beyotime, Shanghai, China) was used. Cells were seeded in a 96-well plate at 5 × 10^3^ cells per well with different drugs for 24 h prior to treatment with DOX. According to the instructions for the CCK-8, 20 μL was added to cells. Then, the cells were incubated in the incubator for 1 h. Finally, the absorbance was measured at 450 nm.

### 2.5. Terminal Deoxynucleotidyl Transferase-Mediated dUTP Nick End Labeling (TUNEL) 

To further verify the degree of apoptosis, TUNEL (Beyotime, Shanghai, China) staining was used. Cells were seeded in a 6-well plate for 48 h with different concentrations of drugs. According to the instructions for the TUNEL, 100 µL was added to cells. Then, the cells were incubated in the incubator for 1 h. Subsequently, PBS was used to remove residual dyes in cells. The ratio of TUNEL-positive cells to the number of nuclei stained by DAPI represents the degree of apoptosis.

### 2.6. Histological Analysis and Immunofluorescence Staining

After the experiments, the mice were anesthetized and their heart tissues were taken out for histological analysis. Immunofluorescence and hematoxylin and eosin (H&E) staining were performed according to previous studies [20].

### 2.7. Reverse-Transcription Quantitative PCR (qPCR)

Total RNA was isolated from heart tissue samples and cells using TRIzol (Takara, Otsu, Japan). cDNA was synthesized using the Prime-ScriptTMRT reagent kit (Takara, Otsu, Japan). In line with directions, we used SYBR Green (Takara, Otsu, Japan) to perform qRT-PCR. GAPDH was used as an internal control. The primer sequences performed in this study for the target genes were as follows:
GAPDH (Mus musculus) F TGCACCACCAACTGCTTAGGAPDH (Mus musculus) R GGATGCAGGGATGATGTTCRRM2 (Mus musculus) F ACTGTGACTTTGCCTGCCTGATGRRM2 (Mus musculus) R TCCGTGAGGAACTCCTGCTCTATC


### 2.8. Western Blot

Extraction of proteins from primary cardiomyocytes and heart tissues was performed with RIPA lysis buffer (Beyotime, Shanghai, China) mixed with protease and phosphatase inhibitors (Beyotime, Shanghai, China). The protein was separated using 7.5–12.5% sodium dodecyl sulfate-polyacrylamide gel electrophoresis (SDS-PAGE) and transferred to a polyvinylidene fluoride (PVDF) membrane. TBST was used to prepare 5% nonfat dry milk for 1 h at room temperature, and then it was incubated overnight with a primary antibody at 4 °C. The antibodies we used were as follows: RRM2 (ABclonal, A5255), Bcl-2 (Proteintech, 12789-1-AP), Phospho-mTOR (CST, 5536T), cleaved-Caspase3 (CST, 9661S), Beclin 1 (ABclonal, A7353), mTOR (CST, 2983T), AKT (Proteintech, 10176-2-AP), Phospho-Akt (Abclonal, AP1259), LC3B (Abclonal, A19665), GAPDH (Abcam, ab181602). Finally, HRP-conjugated secondary antibodies were used at room temperature for 1 h. Signals were visualized by enhanced chemiluminescence ECL (Thermo Scientific, Boston, MA, USA).

### 2.9. Data Analysis

All values are expressed as means ± standard deviation (SD) of independent experiments. Differences between groups were analyzed by an unpaired, two-tailed Student *t*-test (two groups) or ANOVA (three or more groups) followed by Bonferroni’s correction if needed. All of the statistical tests were performed with the GraphPad Prism software version 5.0, and differences with *p* < 0.05 were considered statistically significant. 

## 3. Results

### 3.1. RRM2 Expression Is Decreased In Vitro and In Vivo after DOX Treatment

**Figure 1:**
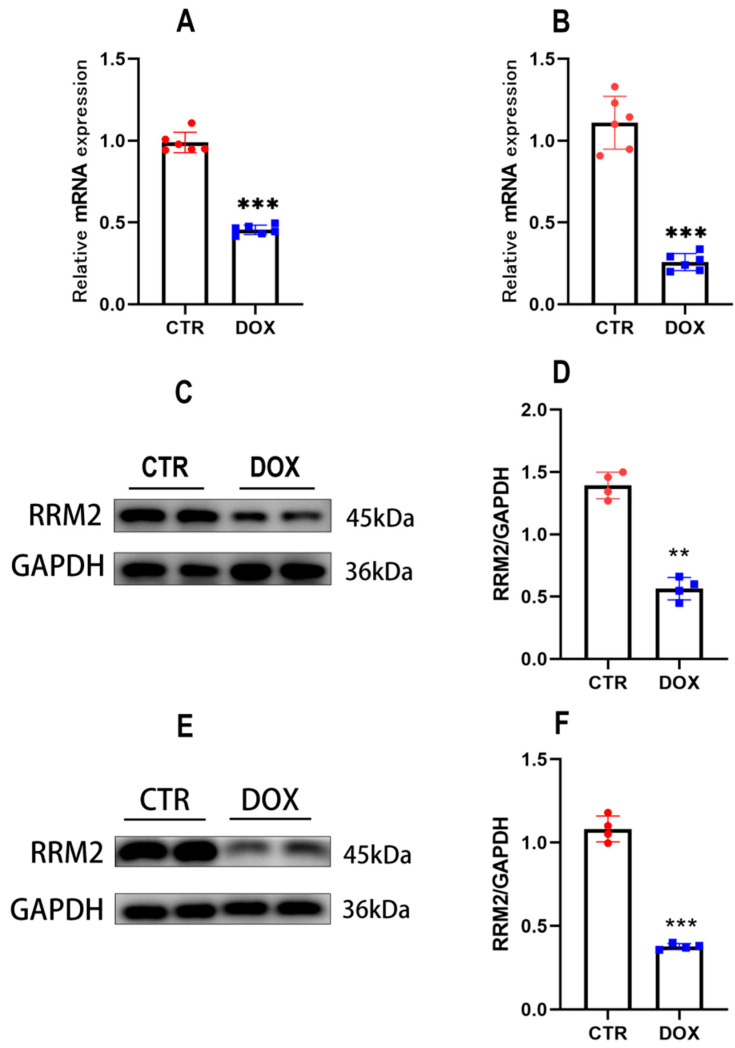
RRM2 expression was decreased in vitro *and* in vivo after DOX treatment. (**A**) Compared with the CTR group, the mRNA expression of RRM2 in primary cardiomyocytes was decreased after DOX treatment (*n* = 6). (**B**) Compared with the CTR group, the mRNA expression of RRM2 in hearts was lower after DOX treatment (*n* = 6). (**C**,**D**) RRM2 protein levels in primary cardiomyocytes treated with DOX (*n* = 4). (**E**,**F**) RRM2 protein levels in hearts 5 days after DOX treatment (*n* = 4). ** indicates *p* < 0.01. *** indicates *p* < 0.001.

Firstly, the effect of DOX on RRM2 expression was detected by qRT-PCR and Western blot. After the cells were treated with DOX (1 µmol/L) for 24 h, we found that the expression of RRM2 mRNA (Figure 1A) and protein levels (Figure 1C,D) were both decreased in primary cardiomyocytes, compared with the control group. RRM2 expression was also investigated in mice after intraperitoneal injection of DOX (15 mg/kg) for 5 days. Similarly, RRM2 mRNA expression (Figure 1B) and protein levels (Figure 1E,F) were markedly downregulated compared with the control group. In summary, these results demonstrated that RRM2 could be implicated in DOX-induced cell damage.

### 3.2. RRM2 Overexpression Abated DOX-Induced Apoptosis and Autophagy In Vitro

**Figure 2:**
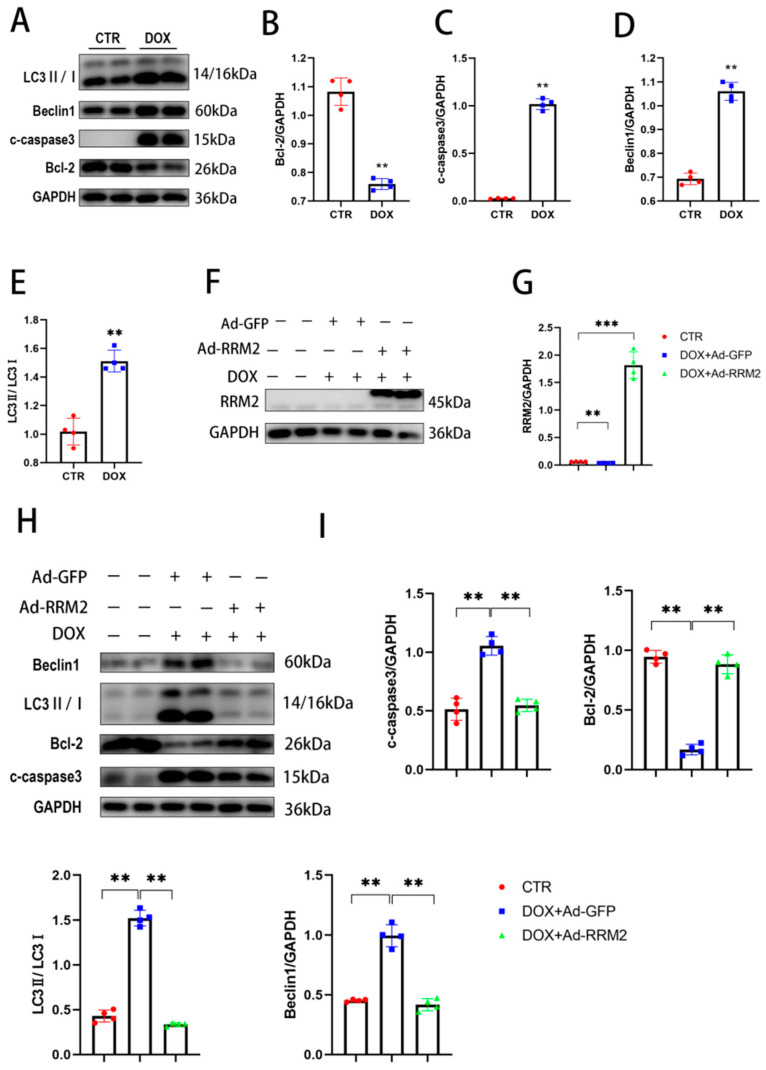
RRM2 overexpression protected against apoptosis and autophagy in cardiomyocytes. (**A**–**E**) The protein expressions of Bcl-2, C-Caspase3, Beclin1, and LC3B in cardiomyocytes after DOX treatment (*n* = 4). (**F**,**G**) Western blot showing RRM2 level in cardiomyocytes after virus transfection (*n* = 4). (**H**,**I**) The protein expression of autophagy and apoptosis (*n* = 4). ** indicates *p* < 0.01. *** indicates *p* < 0.001.

Apoptosis and autophagy are critical to DOX-induced cardiomyopathy [21,22]. To investigate their role, primary cardiomyocytes were treated with DOX for 24 h, and Western blot was used to evaluate the impacts of DOX on primary cardiomyocytes. As shown in Figure 2A–E, DOX treatment prominently increased the protein level of C-Caspase3 and reduced the antiapoptotic protein level of Bcl-2. In addition, autophagy proteins expressions, such as LC3B and beclin1, were significantly increased. Taken together, the mechanism of DOX-induced cardiotoxicity could pertain to the disturbance of apoptosis and autophagy. Subsequently, cardiomyocytes were transfected with overexpressed RRM2 adenovirus (Ad-RRM2) and ctr GFP virus (Ad-GFP) before DOX treatment to further explore the function of RRM2. Western blotting showed that RRM2 was successfully overexpressed after transfection (Figure 2F,G), and its findings confirmed that RRM2 overexpression downregulated the protein levels of C-Caspase3, LC3B, and beclin1—while Bcl-2 was upregulated, as shown in Figure 2H,I. Collectively, these results provide evidence that RRM2 overexpression protects against apoptosis and autophagy in cells.

### 3.3. Knockdown of RRM2 Facilitates DOX-Induced Injury In Vitro

**Figure 3:**
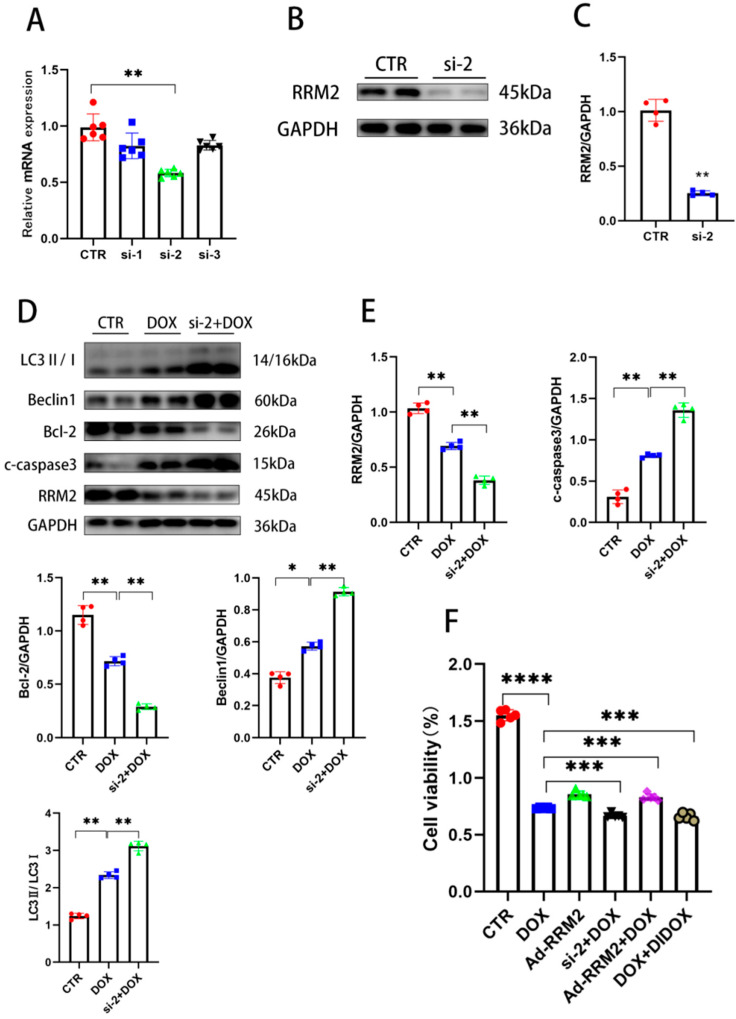
RRM2 knockdown facilitated DOX-induced injury in vitro. (**A**) The mRNA expression of RRM2 after si-RNA transfection (*n* = 6). (**B**,**C**) The protein expression of RRM2 after si-2 transfection (*n* = 4). (**D**,**E**) The protein expression of autophagy and apoptosis after si-2 and DOX treatment (*n* = 4). (**F**) Cell viability after Ad-RRM2, si-2, and DIDOX treatment. (*n* = 5). * indicates *p* < 0.05. ** indicates *p* < 0.01. *** indicates *p* < 0.001. **** indicates *p* < 0.0001.

To investigate whether RRM2 deficiency played a role in DOX-induced cardiotoxicity, si-RNA targeting RRM2 was transfected into cardiomyocytes, and qRT-PCR and Western blot were used to detect the efficiency of si-RNA. The qRT-PCR and Western blot results showed that the mRNA and protein levels of RRM2 were markedly decreased after transfecting si-RNA (Figure 3A,B). Western blot results also confirmed that RRM2 knockdown upregulated the expression of pro-apoptotic and autophagy-related proteins, such as C-Caspase3, LC3B, and beclin1, and downregulated Bcl-2 after DOX treatment (Figure 3D,E). These findings prove that RRM2 deletion could affect apoptosis and autophagy upon DOX treatment. Additionally, it has been reported that RRM2 can affect the proliferation of tumor cells [23]. To detect the effects of RRM2 on cytotoxicity and proliferation, CCK8 was used to verify the function of RRM2 on H9C2 cells. The cells were randomly divided into five groups. Before DOX treatment, three groups were added with DIDOX (RRM2 inhibitor, MCE), si-RNA, Ad-RRM2 for pretreatment, respectively. Our findings demonstrated that RRM2 overexpression could reduce the adverse effects of DOX on cell proliferation, while the inhibition of RRM2 enhanced this effect, as shown in Figure 3F. Altogether, these data demonstrated that RRM2 uniquely affects cell proliferation.

### 3.4. RRM2 Overexpression Alleviated DOX-Induced Cardiotoxicity In Vivo

**Figure 4:**
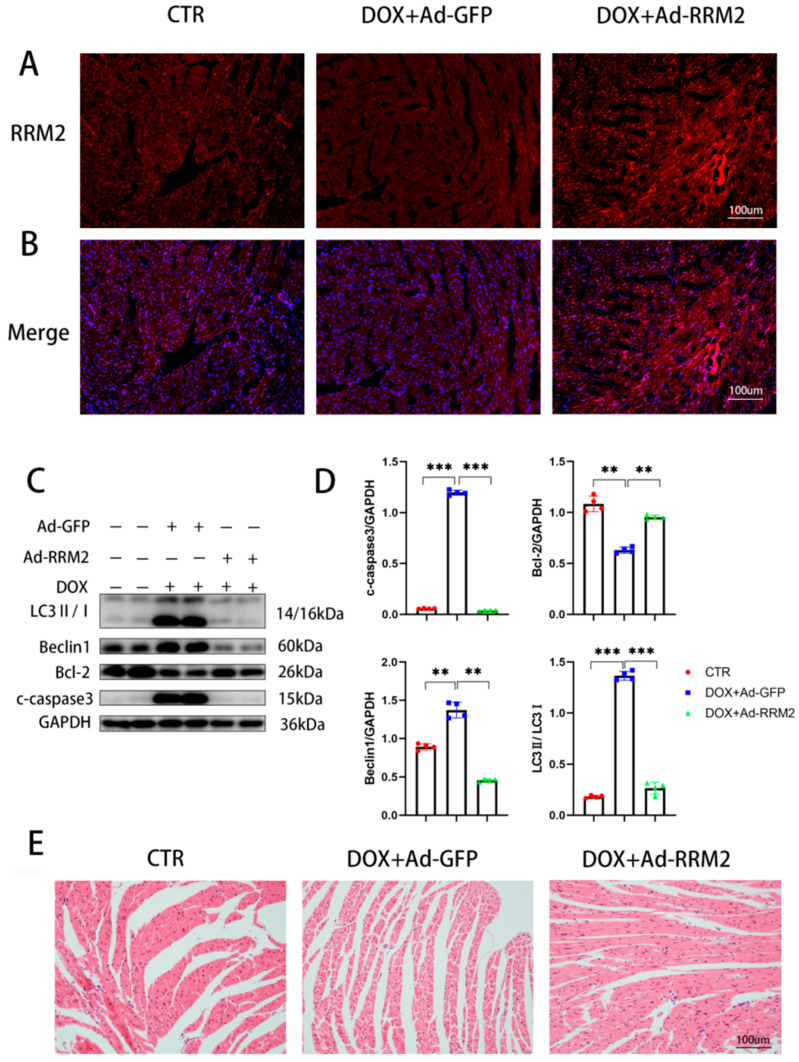
RRM2 overexpression alleviated DOX-induced cardiotoxicity in vivo. (**A**,**B**) Immunofluorescence data revealed that Ad-GFP and Ad-RRM2 were successfully transferred into hearts (*n* = 4). (**C**,**D**) The protein levels of autophagy and apoptosis in mice after DOX treatment (*n* = 4). (**E**) H&E staining in hearts compared with DOX and adenovirus treatments. ** indicates *p* < 0.01. *** indicates *p* < 0.001.

Since DOX treatment could downregulate the levels of RRM2, and as apoptosis and autophagy were aggravated after RRM2 was knocked down, we then verified the effect of RRM2 overexpression on the hearts of mice. Ad-RRM2 and Ad-GFP were separately transduced into cardiac tissue by in situ left ventricular injection. Immunofluorescence confirmed that RRM2 was successfully transferred into hearts (Figure 4A,B). As predicted, RRM2 overexpression ameliorated DOX-induced cardiomyopathy, indicated by reduced pro-apoptotic and autophagy-related proteins (Figure 4C,D) and improved myofibrillar degeneration and disruption (Figure 4E). These findings showed that RRM2 overexpression mitigated DOX-induced cardiotoxicity in hearts.

### 3.5. Blocking RRM2 Overexpression Can Reverse Its Protective Effect

**Figure 5:**
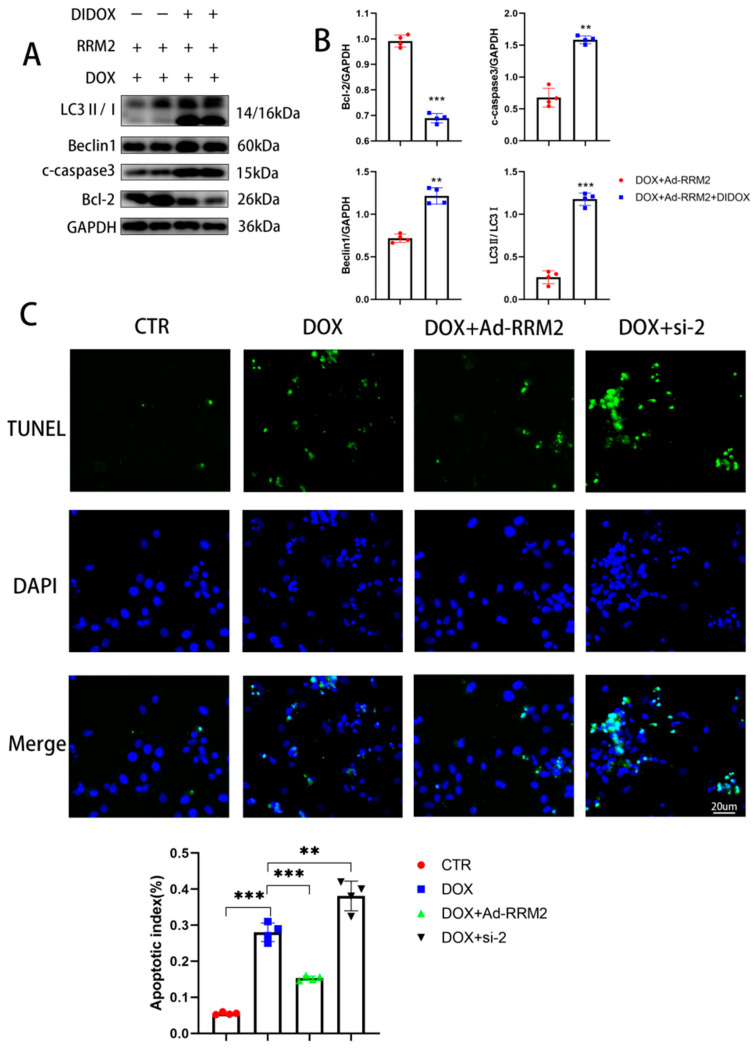
Blocking the overexpression of RRM2 reversed its protective effect. (**A**,**B**) The protein levels of Bcl-2, C-Caspase3, Beclin1, and LC3B in cardiomyocytes after DIDOX and adenovirus treatments (*n* = 4). (**C**) TUNEL staining in each group (*n* = 4). ** indicates *p* < 0.01. *** indicates *p* < 0.001.

To further verify the protective effect of RRM2, we added DIDOX while using adenoviral overexpression of RRM2 to treat cells. The addition of DIDOX (60 µmol/L) during viral transfection blocked the protective effect of RRM2 overexpression, resulting in the increase of LC3B, beclin1, and C-Caspase3 and the decrease of Bcl-2, as shown in Figure 5A,B. In addition, we stained the nucleus with TUNEL (Figure 5C), indicating that the cells had typical nuclear apoptosis. In addition, the apoptotic index was significantly increased when RRM2 was knocked out by siRNA and decreased when RRM2 was overexpressed by adenovirus. In summary, our findings showed that RRM2 improved DOX-induced cardiotoxicity.

### 3.6. AKT/mTOR Signaling Is Involved in Regulating RRM2 in Cardiomyocytes

**Figure 6:**
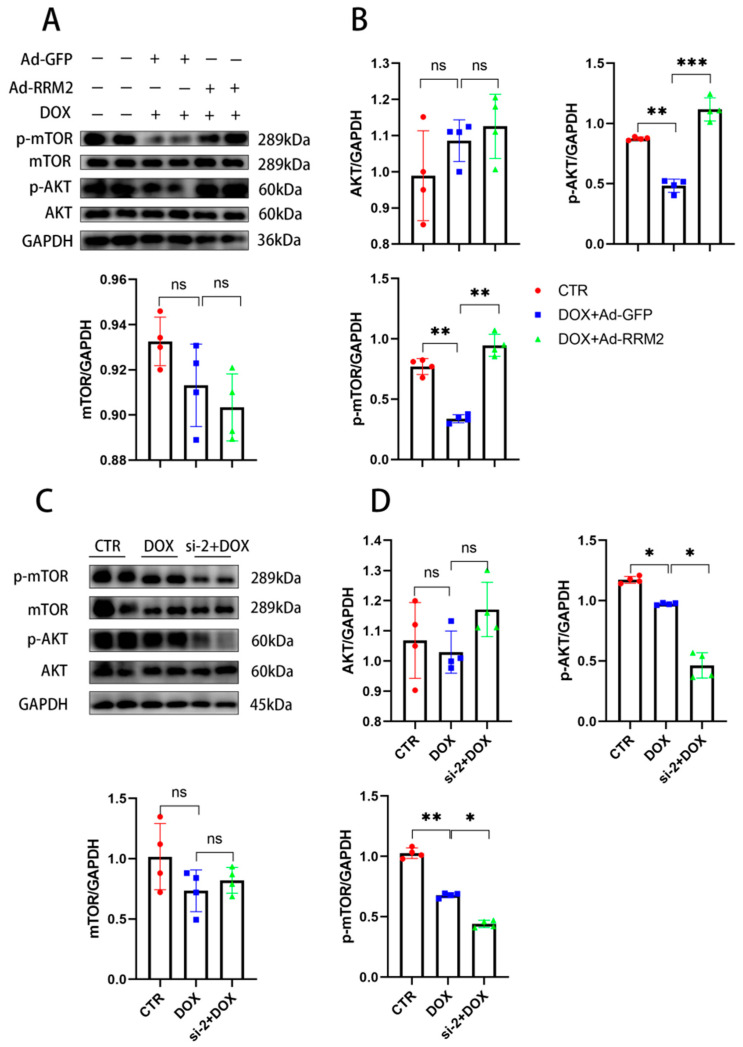
(**A**,**B**) The protein levels of AKT/mTOR signaling pathway in primary cardiomyocytes after DOX and adenovirus treatment (*n* = 4). (**C**,**D**) The protein levels of the AKT/mTOR signaling pathway in primary cardiomyocytes after DOX and si-RNA treatment (*n* = 4). * indicates *p* < 0.05. ** indicates *p* < 0.01. *** indicates *p* < 0.001. ns indicates *p* > 0.05.

**Figure 7:**
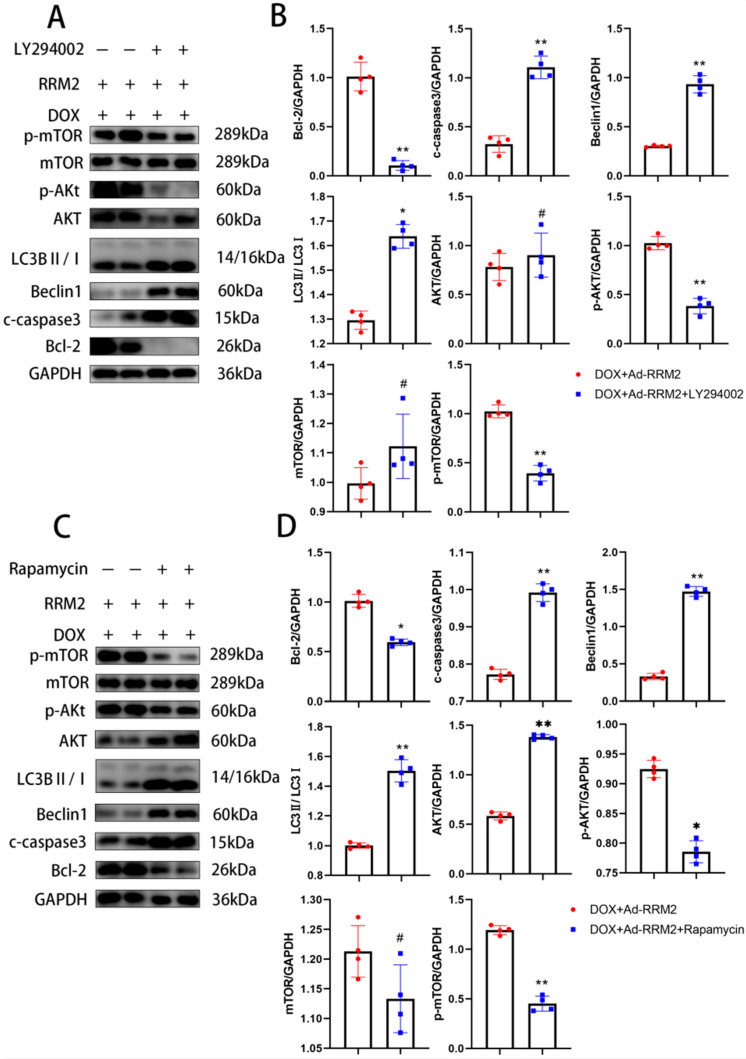
(**A**,**B**) The protein levels of autophagy and apoptosis in primary cardiomyocytes after LY294002 treatment (*n* = 4). (**C**,**D**) The autophagy and apoptosis-related protein levels in primary cardiomyocytes after rapamycin treatment (*n* = 4). * indicates *p* < 0.05. ** indicates *p* < 0.01. # indicates *p* > 0.05.

Current studies revealed that the AKT/mTOR pathway is responsible for DOX-induced cardiomyopathy [24,25]. Consistently, we found that the protein levels of p-AKT and p-mTOR in vitro of the si-RNA or Ad-RRM2 treated groups were significantly changed compared with the DOX group, indicating that the AKT/mTOR signaling pathway was involved in the protective effects of RRM2 on cardiomyocytes (Figure 6). Subsequently, AKT and mTOR inhibitors were used to investigate the role of AKT/mTOR signals in the protection of RRM2 against DOX-induced cardiotoxicity. After treatment with LY294002, an AKT inhibitor, the expression levels of beclin1, LC3B, and C-Caspase3 were markedly upregulated, and Bcl-2 was significantly downregulated in overexpressed RRM2 cardiomyocytes (Figure 7A,B). The same results were obtained when cells were treated with rapamycin, an mTOR inhibitor (Figure 7C,D).

These findings demonstrated that blocking the AKT/mTOR signaling pathway abolished the beneficial effect of RRM2, further confirming that RRM2 has a protective effect against DOX-induced cardiomyopathy through the AKT/mTOR signaling pathway.

## 4. Discussion

**Figure 8:**
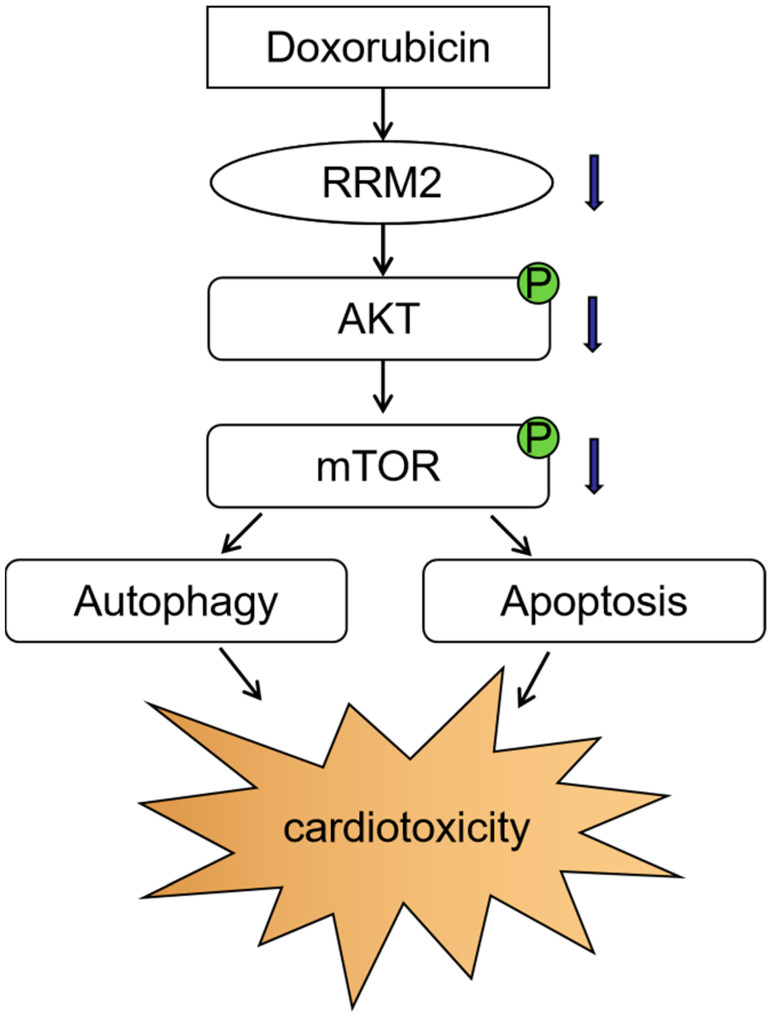
The effects of RRM2 on cardiotoxicity induced by DOX.

Our study revealed the potential protective function of RRM2 on DOX-induced cardiomyopathy and investigated the underlying mechanism (Figure 8). Compared with the control group, RRM2 expression was markedly lower in heart tissues and primary cardiomyocytes after DOX treatment. We also demonstrated that when RRM2 was overexpressed, it could significantly inhibit excessive apoptosis and autophagy—thereby alleviating DOX-induced toxicity—and that this protective effect disappeared after adding RRM2 inhibitors. Consistently, RRM2 knockdown aggravated the development of DOX-induced cardiomyopathy, which could be connected to the disturbance of apoptosis and autophagy. We also found that the protein levels of p-AKT and p-mTOR decreased markedly after DOX treatment, while this effect was reversed after RRM2 overexpression, confirming the AKT/mTOR signaling pathway role in regulating RRM2 in cardiomyocytes. In summary, we believe that RRM2 could be a promising therapy against DOX-induced cardiomyopathy.

At present, it is widely believed that the activity of RR, a nucleotide metabolism enzyme, is closely related to tumor progression. It has been reported that RR can regulate cell proliferation, apoptosis, autophagy, and migration [26]. RR consists of two subunits, RRM1 and RRM2 [27]. Current studies have shown that RRM1 and RRM2 have different effects on tumor progression. For instance, RRM1 knockout could inhibit tumor growth, reduce the risk of metastasis and increase the sensitivity to chemotherapeutic drugs—indicating that RRM1 had pro-tumor functions [28,29]. On the other hand, RRM2 overexpression could significantly enhance the activation potential of multiple oncogenes and increase the risk of malignant tumors [30]. RRM2 is also upregulated in neuroblastoma tissues and is closely related to its clinical stages [15]. Moreover, when the expression of RRM2 is inhibited, it can significantly inhibit cell proliferation and promote apoptosis [31]. In the cell cycle, especially in the S/G2 phase, the transcriptional activation of RRM2 greatly stimulates the activity of RNR to supply the dNTP required for DNA replication [27]. However, there is no current comprehensive mechanism that elucidates the downstream signaling pathway of RRM2 in DOX-induced cardiotoxicity. Our data showed that in vitro and in vivo, DOX-induced cardiotoxicity was significantly reduced when RRM2 was overexpressed, which indicated that RRM2 exerts a protective effect in heart diseases.

DOX is an effective chemotherapeutic drug that plays a key role in the clinical treatment of tumors [32]. However, DOX can cause severe heart damage to patients and even heart failure eventually [33]. Although its clinical effects are remarkable, DOX-related cardiotoxicity is dose-dependent, limiting the promotion of DOX in tumor treatment. The current literature has indicated that many factors participate in the pathogenesis of DOX-induced myocardial damage, such as mitochondrial dysfunction, the disturbance of autophagy, cardiomyocyte apoptosis, and excessive production of ROS [34]. Although multiple mechanisms participate in the evolution of DOX-induced cardiomyopathy, there is currently no method of blocking this process. Therefore, it is urgent that treatment options or potent workarounds are actively explored.

Apoptosis plays a vital role in DOX-induced cardiotoxicity. Existing evidence has shown that both oxidative stress and inflammatory responses induced by DOX could eventually lead to cardiomyocyte apoptosis [35]. This study aimed to analyze the unique function of RRM2 in heart diseases. Previous studies have shown that RR could improve myocardial contractility, and overexpression of RR could significantly enhance the contractility of infarcted hearts [18,36]. These results indicate that RR plays an important role in cardiac function. However, no research on its function in DOX-induced cardiotoxicity has been conducted before. Our results indicate that RRM2 overexpression could inhibit the apoptosis of cardiomyocytes and alleviate heart injury, which could provide a therapeutic option for DOX-induced cardiotoxicity. At present, studies have reported that DOX can cause excessive autophagy, aggravating cell injury [37]. Therefore, some studies have emphasized that reversing DOX-induced excessive autophagy could develop a protective effect [38,39]. In breast cancer cells, RRM2 overexpression could downregulate autophagy levels, leading to the generation of cell resistance [40]. In the present study, we found that autophagy increased after DOX treatment, which is consistent with previous studies, and RRM2 could reverse the disturbance of autophagy by reducing the expression levels of LC3 and beclin1.

The AKT/mTOR signaling pathway plays an important role in cell apoptosis, metabolic regulation, and proliferation. Existing evidence suggests that the AKT/mTOR pathway plays an important role in DOX-induced cardiotoxicity. In addition, the Akt/mTOR pathway was reported to be involved in regulating RRM2 in retroperitoneal liposarcoma [41]. However, the connection between RRM2 and the AKT/mTOR signaling pathway in DOX-induced cardiomyopathy remains unclear. In our study, DOX treatment did reduce p-AKT and p-mTOR expressions, and RRM2 overexpression reversed this phenomenon, accompanied by decreased apoptosis, and autophagy. To further verify this effect, we added LY294002 and rapamycin separately and found that inhibiting AKT and mTOR in vivo increased apoptosis-related and autophagy-related proteins, abolishing the protective effect of RRM2 overexpression.

In conclusion, our study has found that overexpression of RRM2 could reduce DOX-induced myocardial damage and dysfunction by activating the AKT/mTOR signaling pathway, which provides a new option for the clinical treatment of its toxicity-related side effects.

## Data Availability

All data reported are included and represented in the manuscript.
